# High tumor budding is a strong predictor of poor prognosis in the resected perihilar cholangiocarcinoma patients regardless of neoadjuvant therapy, showing survival similar to those without resection

**DOI:** 10.1186/s12885-020-6695-9

**Published:** 2020-03-12

**Authors:** Takahiro Ito, Naohisa Kuriyama, Yuji Kozuka, Haruna Komatsubara, Ken Ichikawa, Daisuke Noguchi, Aoi Hayasaki, Tekehiro Fujii, Yusuke Iizawa, Hiroyuki Kato, Akihiro Tanemura, Yasuhiro Murata, Masashi Kishiwada, Shugo Mizuno, Masanobu Usui, Hiroyuki Sakurai, Shuji Isaji

**Affiliations:** 1 Department of Hepatobiliary Pancreatic and Transplant Surgery, Mie University Graduate School of Medicine, 2-174 Edobashi, Tsu, Mie 514-8507 Japan; 2 Pathology Division, Mie University Hospital, 2-174 Edobashi, Tsu, Mie 514-8507 Japan

**Keywords:** Perihilar cholangiocarcinoma, Tumor budding, Prognostic factor

## Abstract

**Background:**

Tumor budding (TB) is used as an indicator of poor prognosis in various cancers. However, studies on TB in perihilar cholangiocarcinoma are still limited. We examined the significance of TB in resected perihilar cholangiocarcinoma with or without neoadjuvant therapy.

**Methods:**

Seventy-eight patients who underwent surgical resection at our institution for perihilar cholangiocarcinoma from 2004 to 2017, (36 with neoadjuvant therapy), were enrolled in this study. TB was defined as an isolated cancer cell or clusters (< 5 cells) at the invasive front and the number of TB was counted using a 20 times objective lens. Patients were classified into two groups according to TB counts: low TB (TB < 5) and high TB (TB ≥5).

**Results:**

In this 78 patient cohort, high TB was significantly associated with advanced tumor status (pT4: 50.0% vs 22.2%, *p* = 0.007, pN1/2: 70.8% vs 39.6%, *p* = 0.011, M1: 20.8% vs 1.9%) and higher histological grade (G3: 25.0% vs 5.7%, *p* = 0.014). Disease specific survival (DSS) in high TB was significantly inferior compared to that in low TB group (3-y DSS 14.5% vs 67.7%, *p* < 0.001). Interestingly, DSS in high TB showed similar to survival in unresected patients. In addition, high TB was also associated with advanced tumor status and poor prognosis in patients with neoadjuvant therapy. Multivariate analysis identified high TB as an independent poor prognostic factors for DSS (HR: 5.206, *p* = 0.001).

**Conclusion:**

This study demonstrated that high TB was strongly associated with advanced tumor status and poor prognosis in resected perihilar cholangiocarcinoma patients. High TB can be a novel poor prognostic factor in resected perihilar cholangiocarcinoma regardless of neoadjuvant therapy.

## Background

Perihilar cholangiocarcinoma, which is an epithelial cell malignancy localized to the area between the second degree bile ducts and the insertion of the cystic duct into the common bile duct, represents the most common form of cholangiocarcinoma [1, 2]. Although surgical resection remains the only curative treatment for perihilar cholangiocarcinoma, resection is considered a significant challenge for surgeons, and the prognosis of non-resected patients is very poor [3]. However, even if patients undergo curative resection, many patients have cancer recurrence [4]. Predicting poor prognosis and cancer recurrence is an important issue when planning an adequate and effective therapeutic strategy.

Tumor budding (TB) is a histological phenomenon encountered in various cancers typically described as the presence of single cells or clusters in the tumor stroma, and was first described by Imai in Japanese literature [5]. TB is widely used in the field of colorectal carcinoma as a prognostic factor and a correlated factor with advanced stage [6]. In addition, it has been identified as a novel prognostic factor in various types of cancer such as esophageal [7], pancreatic [8], as well as, cancer of the ampulla [9], and gall bladder [10]. With regards to cholangiocarcinoma, three reports on the impact of TB have recently been published [11–13]. Ogino et al. [11] demonstrated that high TB grade was an independent adverse prognostic factor in 195 perihilar cholangiocarcinoma patients by multivariate analysis. Okubo et al. [12] reported the prognostic significance of TB in resected 299 patients with biliary tree carcinoma (intrahepatic: *n* = 47 (16%), extrahepatic: *n* = 144 (48%), gallbladder: *n* = 50 (17%), ampulla: *n* = 58 (19%)). In addition, Tanaka et al. [13] demonstrated that TB tumor budding was a significant prognostic factor in 107 cases of intrahepatic cholangiocarcinoma and 54 cases of perihilar cholangiocarcinoma.

In recent years, neoadjuvant therapy has become a critical treatment for improving the outcomes of various cancers. Additionally, neoadjuvant therapy is becoming the standard of treatment of locally advanced pancreatic adenocarcinoma. However, there are few reports on the impact of neoadjuvant therapy in the patients with cholangiocarcinoma, although the efficacy of neoadjuvant therapy followed by surgery for “unresectable” locally advanced cholangiocarcinoma has been reported [14, 15].

Studies on the relationship of TB and neoadjuvant therapy remain limited. In the field of rectal and esophageal carcinoma, where the use of neoadjuvant therapy is common, there are several reports showing the prognostic significance of high TB in patients who underwent neoajuvant therapy [7, 16, 17]. These studies have reported the association between high TB and poor prognosis in patients underwent neoadjuvant therapy for esophageal carcinoma [7] and rectal carcinoma [16, 17]. In contrast, the three previous studies on the significance of TB in cholangiocarcinoma have excluded patients with neoadjuvant therapy. Due to an expected increase in the number of patients with perihilar cholangiocarcinima who will undergo neoadjuvant therapy followed by curative-intend surgery, we considered which prognostic factors should be analyzed, with additional focus on preoperative treatment. In the present study, we aimed to elucidate the significance of TB in resected perihilar cholangiocarcinoma. In addition, we sought to examine the relationship of TB and neoadjuvant therapy.

## Methods

### Patients

Between January 2004 and December 2017, 81 patients with perihilar cholangiocarcinoma underwent surgical resection at our institution. Three patients with hospital death were excluded, and the remaining 78 patients were included in this study. In addition, 28 patients with locally advanced perihilar cholangiocarcinoma who did not undergo surgical resection in the same period were included in the survival analysis with comparison to resected patients.

### Preoperative management

Multidetector-row computed tomography (MDCT), magnetic resonance cholangiopancreatography (MRCP), endoscopic retrograde cholangiography (ERC), and intraductal ultrasonography (IDUS) were used for preoperative diagnosis and tumor staging. Tumor and negative biopsies by ERC were used for confirming diagnosis and definition of biliary cancer invasion. Endoscopic retrograde biliary drainage (ERBD) tubes were inserted into the future remnant liver in patients with obstructive jaundice.

### Neoadjuvant therapy

The use of neoadjuvant therapy was depended on clinical practice from 2004 to 2009. Chemotherapy or chemoradiotherapy (CRT) was used before and/or after resection. As for chemotherapy, gemcitabine-based regimen in combination with S-1, cisplatin, and 5-FU were used. Radiation therapy (RT) was used as local therapy with a total dose of 36–45 Gy. From 2010, we had introduced a new protocol of preoperative chemotherapy. After evaluation of tumor extension to the hepatic artery (HA), portal vein (PV), and bile duct by preoperative imaging studies, two cycles of chemotherapy with gemcitabine (600 mg/m2 on days 7 and 21) plus S-1 (60 mg/m2 daily on days 1–21 every 4 weeks), followed by surgery, was administrated in patients with locally advanced perihilar cholangiocarcinoma with (1) main, bilateral, or contralateral PV and/or HA invasion with or without possible vascular reconstruction; or (2) invasion of the right side of the umbilical portion and the left side of the origin of the right posterior PV; or (3) regional lymph node metastasis [18–20].

### Surgical procedure

The type of hepatectomy was determined using the following factors: the indocyanine green retention rate at 15 min (ICGR15), the hepatic uptake ratio of 99mTc-GSA scintigraphy at 15 min (LHL15), and the future remnant liver volume using CT volumetry [21]. Right hepatectomy with caudate lobectomy was applied to Bismuth type I, II, and IIIa tumors. Left hepatectomy with caudate lobectomy was applied to Bismuth type IIIb tumors. If a tumor obviously extended over the second order biliary radicles, such as Bismuth type IV tumors, trisectionectomy or central bisectionectomy was selected. In several select patients, extrahepatic bile duct resection without hepatectomywas performed due to poor patient condition, such as older age and insufficient remnant liver function. Portal vein embolization (PVE) was indicated when the future remnant liver volume was estimated as less than 40%. Tumor unresectability was determined by preoperative or intraoperative evaluation of tumor extension to hepatic parenchyma or major vessels, and by insufficient remnant liver function for hepatectomy.

### Histology and assessment of tumor budding

All resected specimens were fixed in 10% buffered formalin and paraffin-embedded (FFPE). FFPE blocks were then sliced into 4-μm-thick sections and stained with haematoxylin and eosin (H&E) for microscopic examination. Primary tumor staging (pT) and regional lymph node metastasis (pN) were defined according to Union for International Cancer Control (UICC) 8th edition. TB was defined as an isolated cancer cell or clusters composed of fewer than 5 cancer cells at the site of tumor invasive front according to previous studies [10–13]. The invasive front was defined as the peripheral to whole primary tumor and in the most severe extended area of tumor to the surrounding tissue according to previous studies. The number of TB was counted in a field measuring 0.785 mm^2^ using a 20 times objective lens by microscopy. The independent two pathologists were blinded to the clinical characteristics of the patients and selected a single field for evaluation, so-called ‘hot-spot’ that would include the maximal budding area for determining the TB count. To find a hot-spot, whole invasive front of tumors were evaluated. The maximal value of tumor budding was defined as TB counts for each tumor. Based on previous studies [10, 12, 13], TB counts were classified into two groups: low TB (TB counts < 5) and high TB (TB counts≥5). In addition, Sub-analysis for comparison between groups with TB counts 5–9 vs TB counts ≥5 was performed. The concordance rate was 94.5%. In disagreement cases, these pathologists discussed the findings and reached a consensus.

### Statistical analysis

Continuous data are expressed as median and range. Continuous and categorical variables were compared using the Mann-Whitney U test and Chi-square test, respectively. Disease-specific survival time (DSS) was calculated from the date of initial treatment to the date of cancer related death or the date of last follow-up if death did not occur. Recurrence free survival time (RFS) was calculated from the date of initial treatment to the date of first recurrence of cancer or the date of last follow-up without recurrence if recurrence did not occur in patients without R2 resection. Patients with R2 resection were excluded from RFS analysis. Survival curves were generated by the Kaplan–Meier method, and differences in survival rates were analyzed using the Log rank test. To identify predictors for disease specific survival, COX hazard regression model with significant variables in univariate analysis was used for multivariate analysis. As prognostic factors, age, gender, preoperative tumor marker (CEA, CA19–9), TMN stage, status of tumor lymphovascular (LV) and perinueral invasion, residual tumor status (non-curative resection), tumor budding status (High TB) were analyzed. All tests were two-sided, and the significance level was *p* < 0.05, and the confidence interval was determined at 95%. All analyses were performed using IBM® SPSS® Statistics version 25 (IBM Corporation, Armonk, NY).

## Results

### Patient overview

Patient demographics of 78 patients are shown in Table 1. Forty-seven were men and 31 were women, with a median age of 69 years (range 39–87 years). Thirty-six patients (46%) received neoadjuvant therapy and 18 patients (23%) underwent PTPE prior to resection. The most common type of liver resection was right hepatectomy with caudate lobectomy (*n* = 27, 35%), followed by left hepatectomy with caudate lobectomy (*n* = 26, 33%). Thirty-eight patients (49%) had tumor with regional lymph node metastasis and 6 patients (8%) had distant metastasis: intrahepatic metastasis (*n* = 3) and extrahepatic metastasis (*n* = 3). Fifty-three patients (68%) achieved R0 resection. Postoperative complication with grade III or more (Clavien-Dindo classification) was occurred in 32 patients (41%) and median (range) postoperative hospital days were 45.5 (13–325) days. Median follow-up time was 2.4 years.

**Table 1:** Patient overview in all 78 patients

Factors	All patients (*n* = 78)
Age (y.o.)	69 (39–87)
Gender (Male / Female)	47 / 31
Neoadjuvant therapy	36
Chemotherapy	25
Chemoradiotherapy	11
PTPE	18
Type of liver resection^a^	
No liver resection (PD, Extra bile duct resection)	5
S1,5,6,7,8	27
S1,4,5,6,7,8	7
S1,2,3,4	26
S1,2,3,4,5,8	5
Others	8
Histological type: G1 / G2 / G3 / others	49 / 18 / 9 / 2
TNM (UICC 8th)	
pT: T0 / T1 / T2 / T3 / T4	1 / 8 / 33 / 12 / 24
pN: N0 / N1 or N2	40 / 38
M1 (Intrahepatic / Extrahepatic)	6 (3 / 3)
Residual tumor status: R0 / R1 / R2	53 / 14 / 11
Postoperative hospital stay (median: days)	45.5 (13–325)
Complication ≥ C-D grade III	32
Postoperative chemotherapy	56

*PTPE* percutaneous transhepatic portal vein embolization, *PD* pancreaticoduodenectomy, *C-D* Clavien-Dindo

^a^Expressed as Couinaud’s hepatic segments resected

### Distribution of TB counts and patients classification according to TB status

**Fig. 1:**
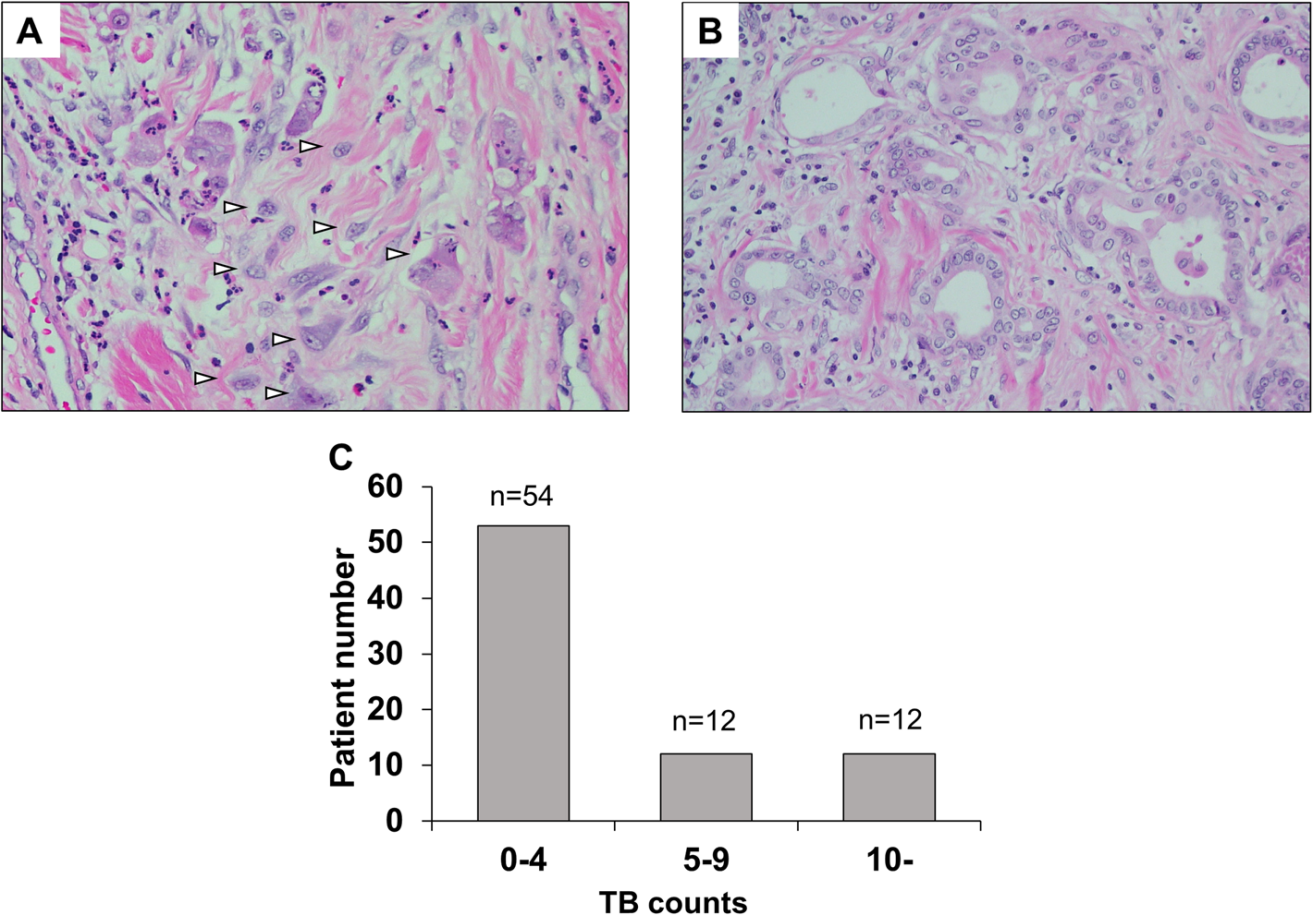
Representative histological findings at the invasive front of tumor and distribution of tumor budding (TB) counts. **a** High TB. **b** Low TB. **c** Distribution of TB counts in the 78 patients. In the front of tumor with high TB, several single cells or clusters (white arrow) are detected (**a**). In contrast, there are few TB in the front of tumor with low TB (**b**). Thirty-one percent of patients (24/78) had tumor with TB counts ≥5 (**c**)

Fig. 1 shows typical histological picture with tumor invasive front with / without tumor budding and distribution of TB counts. Fifty-four patients (69%) had TB counts 0–4, 12 patients (15%) had 5–9, and 12 patients (15%) had 10 or more. Based on some previous studies [7, 8], patients were classified into two groups according to TB; low TB (TB counts < 5, *n* = 54) and high TB (TB counts ≥5, *n* = 24). Then we compared patient characteristics and outcomes.

### Tumor budding was associated with advanced histological status and poor prognosis

As shown in Table 2, there were no significant differences in preoperative patient characteristics and surgical information such as age, gender, preoperative treatment, tumor markers, types of surgery. However, in high TB patients, the rate of patients who underwent combined vascular resection (HA and/or PV) was tended to be lower than that in low TB patients despite it was not statistically significant (67% vs. 43%, *p* = 0.050). In terms of histologically factors, high TB patients had higher rates of tumor with grade G3 (25% vs. 5.6%, *p* = 0.013), pT4 (50.0% vs. 22.2%, *p* = 0.014), lymph node metastasis (70.8% vs. 38.9%, *p* = 0.009), and distant metastasis (20.8% vs. 1.9%, *p* = 0.004). There were no significant differences in postoperative factors such as length of postoperative hospital stay, complication and adjuvant therapy.

**Table 2:** Patient characteristics in the low and high TB groups

Variables	Low TB (*n* = 54)	High TB (*n* = 24)	*p* value
Age (y.o.)	69 (40–87)	69 (39–78	0.630
Gender (Male/Female)	31 / 23	16 / 8	0.441
Neoadjuvant therapy	25 (46.3%)	11 (45.8%)	0.970
PTPE	12 (22.2%)	6 (25.0%)	0.788
Initial tumor marker			
CEA (ng/mL)	3.6 (0.6–38.4)	3.6 (0.7–28.0)	0.862
CA19–9 (U/mL)	76.6 (1.0–7898)	106.6 (1.0–9278)	0.681
Preoperative tumor marker			
CEA (ng/mL)	3.0 (0.7–32.2)	3.2 (0.5–32.3)	0.492
CA19–9 (U/mL)	56.9 (1.0–11,659)	89.7 (1.0–9278)	0.174
Type of liver resection			0.109
No liver resection	5 (9.3%)	0	
S1,5,6,7,8	16 (29.6%)	10 (41.7%)	
S1,4,5,6,7,8	19 (35.2%)	8 (33.3%)	
S1,2,3,4	3 (5.6%)	2 (8.3%)	
S1,2,3,4,5,8	3 (5.6%)	4 (16.7%)	
Others	8 (14.8%)	0	
HPD	3 (5.6%)	1 (4.2%)	0.797
Combined HA and/or PV resection	23 (42.6%)	16 (66.7%)	0.050
Operation time (min)	603 (383–1030)	622 (422–972)	0.482
Blood loss (ml)	2165 (166–9907)	2054 (587–6012)	0.987
**Histological type**			
**G1–2 / G3**	**51 / 3 (5.6%)**	**18 / 6 (25%)**	**0.013**
**TNM (UICC8th)**			
**pT: T0–3 / T4**	**42 / 12 (22.2%)**	**12 / 12 (50.0%)**	**0.014**
**pN: N0 / N1–2**	**33 / 21 (38.9%)**	**7 / 17 (70.8%)**	**0.009**
**M: M0 / M1**^**a**^	**53 / 1 (1.9%)**	**19 / 5 (20.8%)**	**0.004**
Residual tumor status			
R0 / R1 / R2 (M1/ margin positive)	38 / 11 / 5 (1 / 4)	15 / 3 / 6 (5 / 1)	0.162
Postoperative hospital stay (days)	45.5 (13–61)	47.5 (17–325)	0.931
Postoperative complication ≥ grade III^b^	22 (40.7%)	10 (41.7%)	0.939
Postoperative chemotherapy	37 (68.5%)	19 (79.2%)	0.335

There were no significant differences in preoperative patient characteristics and surgical information. In High TB group, patients had higher rate of tumor with grade G3 (25% vs 5.6%, *p* = 0.013), T4 (50.0% vs 22.2%, *p* = 0.014), Lymph node metastasis (70.8% vs 38.9%, *p* = 0.009), and distant metastasis (20.8% vs 1.9%, *p* = 0.004)

*HPD* hepatopancreaticoduodenectomy, *HA* hepatic artery, *PV* portal vein

^a^M1 include 3 patients with intrahepatic metastasis. ^b^Clavien-Dindo classification

**Fig. 2:**
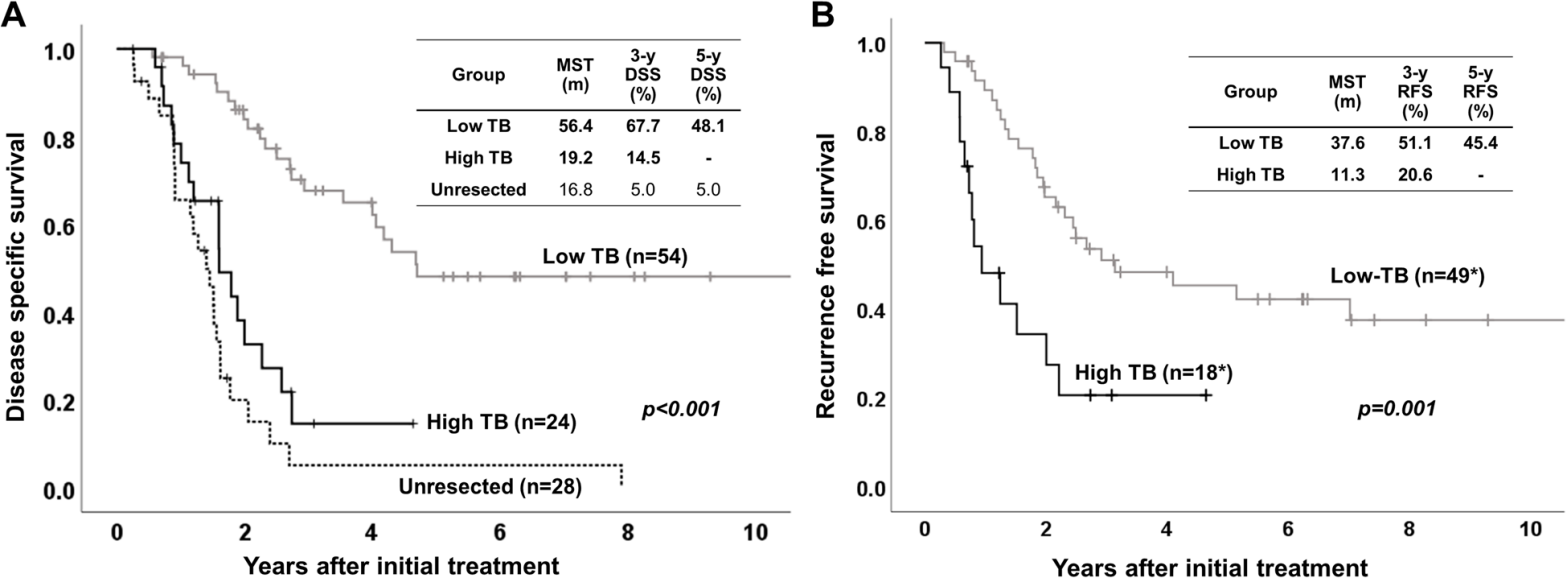
Patient survival according to tumor budding. **a** Disease specific survival. **b** Recurrence free survival. On both disease specific survival (DSS) and recurrence free survival (RFS), patients in high TB group had significantly poor survival compared to patients in low TB group (*p* < 0.001 in DSS, *p* = 0.001 in RFS). Interestingly, DSS in high TB group did not show statistical difference compared to that in 28 unresected patients at our institution in the same period (*p* = 0.103). *Eleven patients with R2 resection (distant metastasis or cancer positive surgical margin) were excluded from RFS study

As for survival, high TB patients had significantly poor as compared to low TB patients on both of DSS and RFS (*p* < 0.001 in DSS, *p* = 0.001 in RFS, Fig. 2). From RFS study, 11 patients with R2 resection (distant metastasis or cancer positive surgical margin) were excluded. Median survival time (MST) of DSS in high and low TB patients was 19.2 and 56.4 months, respectively. MST of RFS in high and low TB patients was 11.3 and 37.6 months, respectively. Interestingly, DSS after initial treatment in high TB patients did not show statistical difference compared to that in 28 unresected patients having locally advanced tumor at our institution in the same period. When we compared DSS and RFS between patients with TB5–9 and those with TB 10 or more, there were no differences between two groups (Figure S).

### Tumor budding was associated with advanced histological features and poor survival in patients with neoadjuvant therapy

**Fig. 3:**
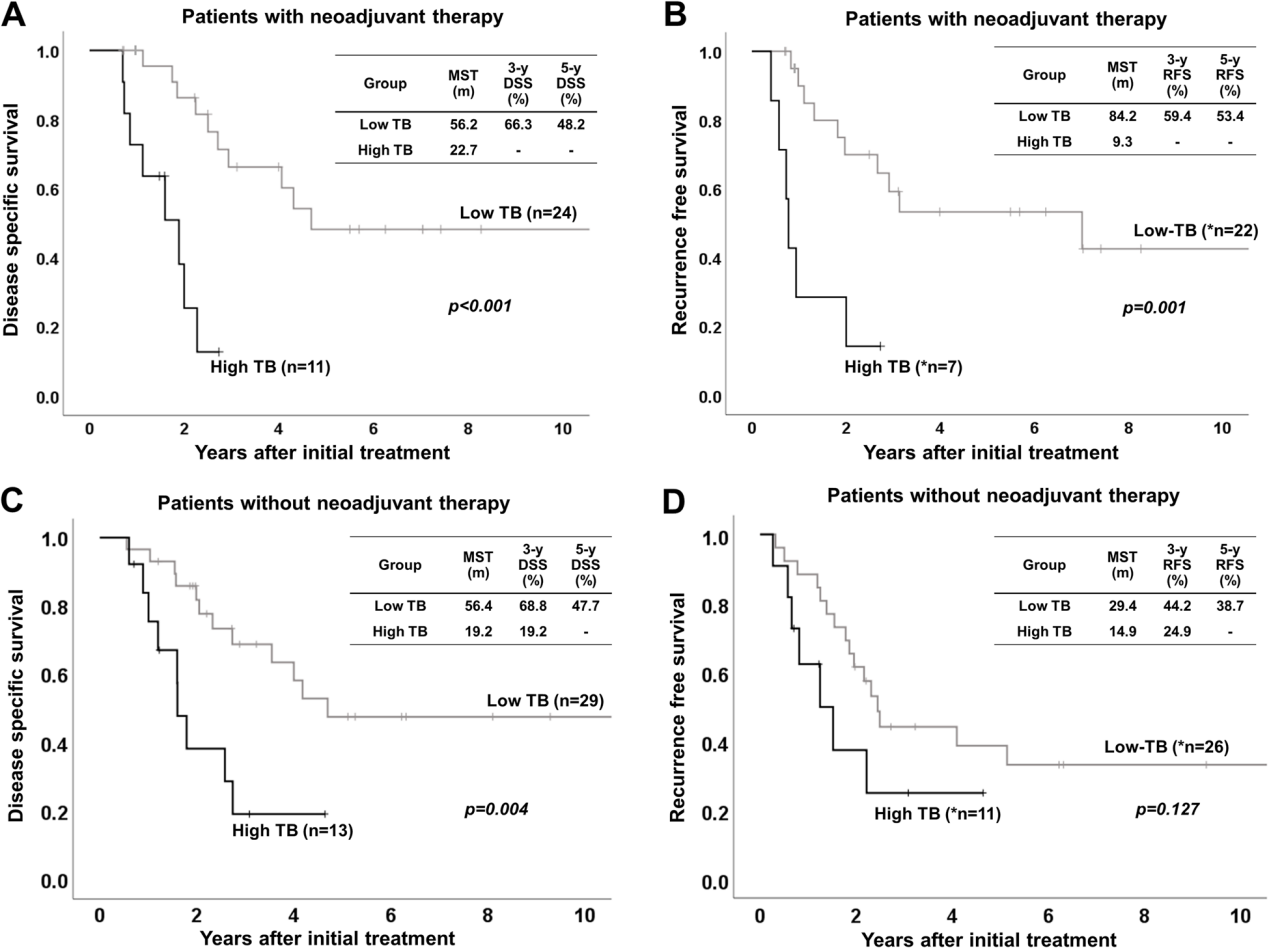
Patient survival according to tumor budding in patients with or without neoadjuvant therapy. **a, b** Disease specific survival (DSS) and Recurrence free survival (RFS) in patients with neoadjuvant therapy. **c, d** DSS and RFS in patients without neoadjuvant therapy. In patients with neoadjuvant therapy, patients in high TB group had significantly poor survival as compared to patients in low TB group (*p* < 0.001 in DSS, *p* = 0.001 in RFS). Similarly, in patients without neoadjuvant therapy, patients in high TB group had significantly poor DSS and had a tendency with poor RFS, as compared to patients in low TB group (*p* = 0.004 in DSS, *p* = 0.127 in RFS). *6 patients with neoadjuvant therapy and 5 patients without neoadjuvant therapy with R2 resection (distant metastasis or cancer positive surgical margin) were excluded from RFS study

To confirm the significance of TB in the patients who received neoadjuvant therapy (Table 3), we classified the 36 patients with neoadjuvant therapy into low TB (*n* = 25) and high TB (*n* = 11), and classified the 42 patients without neoadjuvant therapy into low TB (*n* = 29) and high TB (*n* = 13). Among the patients with neoadjuvant therapy, high TB patients had a significantly higher rate of combined vascular resection (90.9% vs. 48.0%, *p* = 0.015) compared to low TB patients. In the patients without neoadjuvant therapy, there were no significant differences in pre- and intra-operative factors. In the patients with neoadjuvant therapy, high TB patients, as compared to low TB patients, had significantly higher rates of G3 (45.5% vs. 0%, *p* < 0.001), pT4 (63.6% vs. 24.0%, *p* = 0.023), lymph node metastasis (72.7% vs. 32.0%, *p* = 0.023), and distant metastasis (27.3% vs. 0%, *p* = 0.006). As for postoperative factors, there were no differences between the two groups. Figure 3 shows patients survival according to TB status in patients with or without neoadjuvant therapy. In the patients with neoadjuvant therapy, high TB patients had significantly poor survival as compared to low TB patients (*p* < 0.001 in DSS, *p* = 0.001 in RFS). In the patients without neoadjuvant therapy, high TB patients had significantly poor DSS, as compared to Low TB patients, but RFS had no significantly difference between two groups.

**Table 3:** Characteristics in the patients with or without neoadjuvant therapy

	Patients with neoadjuvant therapy (*n* = 36)			Patients without neoadjuvant therapy (*n* = 42)		
Factors	Low TB (*n* = 25)	High TB (*n* = 11)	*p* value	Low TB (*n* = 29)	High TB (*n* = 13)	*p* value
Age (y.o.)	70 (49–84)	69 (39–77)	0.520	67 (40–87)	69 (44–78)	> 0.999
Gender (Male / Female)	13 / 12	9 / 2	0.091	18 / 11	7 / 6	0.616
Type of Neoadjuvant therapy						
Chemotherapy	17 (68.0%)	8 (72.7%)	0.777	–	–	–
Chemoradiotherapy	8 (32.0%)	3 (27.3%)				
PTPE	4 (16.0%)	2 (18.2%)	0.871	8 (27.6%)	4 (30.8%)	0.833
Initial tumor marker						
CEA (ng/mL)	3.7 (1.3–14.6)	3.9 (0.9–16.3)	0.685	2.9 (0.6–38.4)	2.6 (0.7–28.0)	0.936
CA19–9 (U/mL)	61.5 (1.0–7898)	140.3 (1.0–1325)	0.435	87.5 (7.0–1115.7)	65.1 (1.0–9278)	0.872
Preoperative tumor marker						
CEA (ng/mL)	2.6 (0.9–9.6)	3.6 (1.1–24.4)	0.151	3.0 (0.7–32.2)	2.6 (0.5–32.3)	0.788
CA19–9 (U/mL)	33.2 (1.0–11,659)	151.3 (1.0–1158)	0.086	67.3 (13.7–977.2)	65.1 (1.0–9278)	0.936
Type of liver resection^a^						0.114
No liver resection	0	0		5 (17.2%)	0	
S1,5,6,7,8	9 (36.0%)	3 (27.3%)		10 (34.5%)	5 (38.5%)	
S1,4,5,6,7,8	1 (4.0%)	1 (9.1%)		2 (6.9%)	3 (23.1%)	
S1,2,3,4	9 (36.0%)	6 (54.5%)		7 (24.1%)	4 (30.8%)	
S1,2,3,4,5,8	3 (12.0%)	1 (9.1%)		0	1 (7.7%)	
Others	3 (12.0%)	0		5 (17.2%)	0	
HPD	0	1 (9.1%)	0.126	3 (10.3%)	0	0.229
**Combined HA and/or PV resection**	12 (48.0%)	10 (90.9%)	**0.015**	11 (37.9%)	6 (46.2%)	0.616
Operation time (min)	625 (383–965)	672 (422–972)	0.261	597 (403–1030)	610 (435–746)	0.914
Blood loss (ml)	2212 (505–6916)	2170 (1459–6012)	0.612	1964 (166–9907)	1830 (587–3870)	0.727
**Histological type**						
**G1, 2 / G3**	25 / 0 (0%)	6 / 5 (45.5%)	**< 0.001**	26 / 3 (10.3%)	12 / 1 (7.7%)	0.787
UICC 8th						
**pT: T0–3 / T4**	19 / 6 (24.0%)	4 / 7 (63.6%)	**0.023**	23 / 6 (20.7%)	8 / 5 (38.5%)	0.226
**pN: N0 / N1–2**	17 / 8 (32.0%)	3 / 8 (72.7%)	**0.023**	16 /13 (44.8%)	4 / 9 (69.2%)	0.143
**M: M0 / M1**^**a**^	25 / 0 (0%)	8 / 3 (27.3%)	**0.006**	28 / 1 (3.4%)	11 / 2 (15.4%)	0.165
Residual tumor status						
R0 / R1 / R2 (M1 / margin positive)	17 / 6 / 2 (0 / 2)	6 / 1 / 4 (3 / 1)	0.092	21 / 5 / 3 (1 / 2)	9 / 2 / 2 (2 / 0)	0.895
Postoperative hospital stay (days)	45 (13–161)	37 (17–136)	0.308	49 (25–117)	56 (18–325)	0.374
Postoperative complication ≥ grade III^b^	13 (52.0%)	6 (54.5%)	0.888	9 (31.0%)	4 (30.8%)	0.986
Postoperative chemotherapy	19 (76.0%)	9 (81.8%)	0.699	18 (62.1%)	10 (76.9%)	0.345

Patients in high TB groups had a significantly higher rate of combined vascular resection in patients received neoadjuvant therapy. In patients without preoperative therapy, there were no significant differences in preoperative and operative factors. Among 36 patients with neoadjuvant therapy, the rates of G3, T4, N1–2, and M1 in high TB group were significantly higher than those in low TB. As for postoperative factors, there were no differences between two groups in both patients with and without neoadjuvant therapy

^a^M1 include 3 patients with intrahepatic metastasis. ^b^ Clavien-Dindo classification

### High tumor budding was an independent poor prognostic factor by multivariate analysis for disease specific survival

**Fig. 4:**
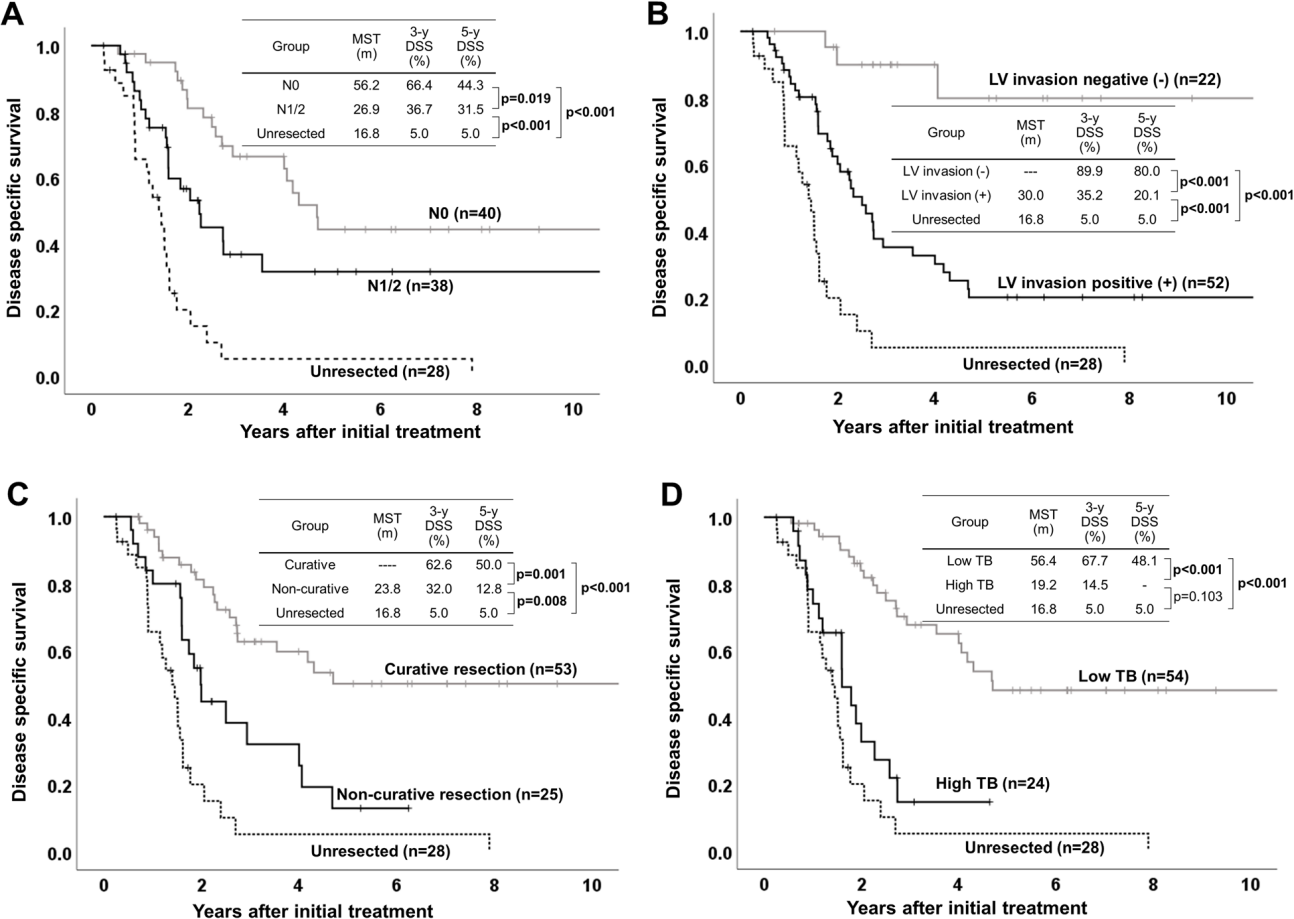
Disease specific survival according to independent prognostic factors in the 78 patients. **a** N1/2 vs N0. **b** LV invasion positive (+) vs LV invasion negative (−). **c** Non-curative resection vs curative resection **d** High TB vs Low TB. In all comparison according to each independent prognostic factor, patients with each factor had significantly poor survival compared to those without it. DSS in patients with high TB did not show significantly difference compared to DSS in unresected patients

To identify predictors for DSS and to confirm the significance of TB, multivariate COX regression analysis was performed. As shown in Table 4, pre-operative CEA level (≥ 5 ng/ml), histological grade G3, T4, N1/2, M1, LV invasion, non-curative resection, and High TB, were identified as poor prognostic factors for DSS by univariate analysis. Multivariate analysis identified N1/2, LV invasion, non-curative resection, and High TB, as independent significant poor prognostic factors for DSS. In Fig. 4, we compared DSS according to independent prognostic factors in the 78 patients. In all comparison according to each factors (N1/2, LV invasion, non-curative resection, and high TB), patients with each prognostic factors had significantly inferior survival compared to those without it. Among four patient classifications, notably, DSS in only patients with high TB did not show significantly difference compared to DSS in 28 unresected patients.

**Table 4:** Uni- and multivariate analysis for poor disease specific survival

Factors	Univariate analysis		Multivariate analysis	
	Hazard Ratio (95% CI)	*p* value	Hazard Ratio (95% CI)	*p* value
Patient age (≥70 years)	0.742 (0.385–1.430)	0.373		
Gender (male)	0.779 (0.408–1.486)	0.779		
Pre-operative CEA level (≥ 5 ng/ml)	**2.071 (1.021–4.199)**	**0.044**	0.531 (0.241–1.319)	0.173
Pre-operative CA 19–9 level (≥ 100 U/ml)	1.479 (0.767–2.852)	0.243		
Histological grade: G3	**3.350 (1.514–7.414)**	**0.003**	1.145 (0.433–3.025)	0.785
T stage: T4	**2.366 (1.258–4.452)**	**0.008**	1.221 (0.598–2.492)	0.584
N stage: N1 or N2	**2.111 (1.115–3.994)**	**0.022**	**2.354 (1.010–5.487)**	**0.047**
M stage: M1	**9.524 (3.434–26.411)**	**< 0.001**	1.655 (0.481–5.689)	0.424
Lymphovascular invasion	**7.654 (2.349–24.937)**	**0.001**	**5.307 (1.530–18.413)**	**0.009**
Perinueral invasion	28.161 (0.546–1451.390)	0.097		
Non-curative resection	**2.792 (1.471–5.299)**	**0.002**	**2.456 (1.116–5.408)**	**0.026**
High TB	**4.493 (2.276–8.870)**	**< 0.001**	**5.206 (1.985–13.655)**	**0.001**

Regional lymph node metastasis, lyphovascular invasion, non-curative resection, and High TB were identified as independent poor prognostic factors for DSS

## Discussion

In order to improve prognosis and implementation of therapeutic strategies for patients with perihilar cholangiocarinoma, it is crucial to identify new significant prognostic factors. In the present study, we first elucidated the prognostic significance of high TB (TB counts ≥5) at the tumor invasive front by analyzing our patient database, including approximately half of patients having received neoadjuvant therapy. In all patients, high TB was significantly associated with advanced tumor status including rates of pT4, pN1/2, M1, and histological grade 3. Survival in patients with high TB was significantly inferior than that in patients with low TB. By multivariate analysis, high TB was identified as one of independent poor prognostic factors for DSS among 4 factors including regional lymph node metastasis, LV invasion, and non-curative resection. Interestingly, DSS in high TB group did not show statistical difference compared to that in unresected patients. In addition, the impact of high TB in patients with neoadjuvant therapy showed similar results, withhigh TB significantly associated with advanced tumor status and poor prognosis.

Many studies have reported several prognostic factors, such as presence of higher histological grade (G3), higher T stage, lymph node metastasis, and positive surgical resection margin, associated with poor survival in resected patients with cholangiocarcinoma [3, 22–24]. In the previous study on TB in extrahepatic cholangiocarcinoma, Ogino, et al. [11] demonstrated high TB as an independent adverse prognostic factor in multivariate analysis, along with higher T stage, lymph node metastasis, and resected margin positive invasive carcinoma. The present study similarly showed that high TB, N1/2, LV invasion, and non-curative resection were identified independent poor prognostic factors in all patients. Therefore, high TB has potential to be a new pathological prognostic factor.

Evaluation of TB can easily provide useful feedback on the patient’s clinical situation, which can then be easily disseminated from pathologist to clinical physician, because it can be examined in the H&E-stained specimens as a part of conventional pathologic diagnosis. In the present study, the number of TB was counted in a field measuring 0.785 mm^2^ using a 20 times objective lens by microscopy. The pathologist then decided on a “hot-spot” location and calculated the TB counts, that were classified into two groups by using 5 as a cut-off value. As for cut-off value, Okubo et al. classified patients according to ≥5 or < 5, whereas Ogino et al. [11] classified TB into three grades: low-grade, 0–4 TB; intermediate-grade, 5–11 TB; high-grade, TB. Meanwhile, in colorectal cancer and pancreatic ductal adenocarcinoma, other methods for evaluating evaluate TB has been reported [8, 25]. Several reports used immunohistochemistry by cytokeratin to easily identify TB at stroma [8, 25]. Okubo et al. [12] demonstrated the strong correlation between TB counts cytokeratin-stained tissue and the H&E-stained tissue sections in cholangiocarcinoma. In colorectal cancer, evaluation of TB in only H&E stained tissue is widely recognized and performed [6]. Therefore, to easily evaluate TB, we considered using H&E stained tissue as a prognostic factor. In the present study, there were no differences in DSS and RFS between patients with TB5–9 and those with TB 10 or more, although further studies for cut-off value and method of counting are warranted.

Many previous studies on various malignant tumors have reported a correlation between high TB and advanced tumor. In cholangiocarcinoma, Ogino, et al. [11] reported similar results to the current study: that the high TB grade was associated with poor histological differentiation, higher pT factor, regional lymph node metastasis, and a higher rate of residual invasive tumor in the resected margin. They considered that TB at the tumor invasive front may cause cancer cell migration through the extracellular matrix, invade lymphovascular structures, and represent the first step towards cancer metastasis. To progress to this point, cancer cells need to change their own phenotype in a process known as, epithelial-mesenchymal transition (EMT), which is a multistep dynamic cellular phenomenon in which epithelial cells lose their cell–cell adhesion and gain migratory and invasive traits that are typical of mesenchymal cells [26]. In several reports, TB was found to be associated with EMT [11, 27]. In addition, Ogino et al. [11] have confirmed the correlation between TB and EMT in cholangiocarinoma, demonstrating that TB counts are significantly higher in EMT status in TB; the low-expression of E-cadherin (epithelial marker) and high-expression of Vimentin (mesenchymal marker).

A noteworthy point of the present study, is the demonstration of the prognostic significance of TB in patients with neoadjuvant therapy. There are several reports showing the significance of high TB in patients who underwent neoajuvant therapy [7, 16, 17] for rectal and esophageal carcinoma. Miyata et al. [7] showed that TB in the invasive front of tumors was significantly correlated with prognosis in 74 patients who received neoadjuvant chemotherapy for advanced esophageal squamous cell carcinomas. In their study, they discussed the mechanisms of TB formation. They speculated that TB in tumor received neoadjuvant chemotherapy for esophageal cancers may represent cross-interaction between bone marrow-derived cells and cancer cells in the invasive front. Several in vitro studies demonstrated that bone marrow-derived cells, which are recruited to the tumor invasion front through chemokine signaling, promote tumor invasion and metastasis [28, 29]. In another study on the prognostic value of tumor budding in rectal cancer after neoadjuvant radiotherapy, Du et al. [16] demonstrated that high grade TB was the major factor affecting 5-year RFS. Meanwhile, Sannier et al. [17] chose a more easily applicable technique for evaluation of TB in patients who received neoadjuvant chemoradiotherapy for locally advanced rectal carcinoma without any cut off. Consequently, the presence of TB, even in low numbers, is considered to have an adverse effect on outcome. In our present study, there were no differences in TB counts between patients with or without neoadjuvant therapy. However, further studies are needed to clarify the mechanism of TB formation in patients with neoadjuvant therapy.

Interestingly, DSS in resected patients with high TB did not show a significant difference compared to that in unresected patients, suggesting that they may not have better prognosis irrespective of whether they can achieve R0 resection. For these patients, we should consider the necessity of additional peri-operative therapy. The TB evaluation method employed in the present study, a pathologist determined “hotspot,” is limited in that it cannot evaluate TB before surgery. Therefore, there is an urgent need to identify preoperative predictors of high TB and establish new therapeutic strategies. This should include improving surgical technique, as well as, developing effective new preoperative and postoperative adjunctive therapy.

Our present study included several limitations. This study was retrospective study with a small number of patients. In addition, the indications and types of neoadjuvant therapy in each patient were not uniform. However, it is noteworthy that TB was strongly associated with poor prognosis even in small number cohort. Additional prospective studies are warranted.

## Conclusion

Our present study demonstrated that high TB at the invasive front of tumors in resected perihilar cholangiocarcinoma patients with or without neoadjuvant therapy, is strongly associated with advanced tumor status and poor prognosis, including DSS/RFS. High TB could be a novel prognostic factor in resected perihilar cholangiocarcinoma even if patients received neoadjuvant therapy.

## Supplementary information

**Additional file 1: Figure S.** Disease specific survival (DSS) and recurrence free survival (RFS) according to TB counts. Both of DSS (A) and RFS (B) did not show differences between patients with TB 5–9 and those with TB 10 or more.

## Data Availability

The datasets used and/or analyzed during the current study are available from the corresponding author on reasonable request.

## Abbreviations

CRT: Chemoradiotherapy
DSS: Disease-specific survival time
EMT: Epithelial-mesenchymal transition
ERBD: Endoscopic retrograde biliary drainage
ERC: Endoscopic retrograde cholangiography
HA: Hepatic artery
IDUS: Intraductal ultrasonography
IRB: Institutional Review Board
LV: Lymphovasclular
MDCT: Multidetector-row computed tomography
MRCP: Magnetic resonance cholangiopancreatography
MST: Median survival time
PV: Portal vein
PVE: Portal vein embolization
RFS: Recurrence free survival time
RT: Radiation therapy
TB: Tumor budding
UICC: Union for International Cancer Control
