# Supplementary material for: High tumor budding is a strong predictor of poor prognosis in the resected perihilar cholangiocarcinoma patients regardless of neoadjuvant therapy, showing survival similar to those without resection
Source: BMC Cancer. 2020 Mar 12;20:209. doi: 10.1186/s12885-020-6695-9 (PMC7069056; doi:10.1186/s12885-020-6695-9)
Supplement: Supplementary file 1 — Additional file 1: Figure S. Disease specific survival (DSS) and recurrence free survival (RFS) according to TB counts. Both of DSS (A) and RFS (B) did not show differences between patients with TB 5–9 and those with TB 10 or more. [file 12885_2020_6695_MOESM1_ESM.pdf]

Supplementary materials

High tumor budding is a strong predictor of poor prognosis in the resected perihilar cholangiocarcinoma patients regardless of neoadjuvant therapy, showing survival similar to those without resection

Authors:

Takahiro Ito[1], Naohisa Kuriyama[1], Yuji Kozuka[2], Haruna Komatsubara[2],Ken Ichikawa[1], Daisuke Noguchi[1], Aoi Hayasaki[1], Tekehiro Fujii[1], Yusuke Iizawa[1], Hiroyuki Kato[1], Akihiro Tanemura[1], Yasuhiro Murata[1], Masashi Kishiwada[1], Shugo Mizuno[1], Masanobu Usui[1], Hiroyuki Sakurai[1], and Shuji Isaji[1]

Institution:

1) Department of Hepatobiliary Pancreatic and Transplant Surgery, Mie University Graduate School of Medicine, 2-174 Edobashi, Tsu, Mie 514-8507, Japan
2) Pathology Division, Mie University Hospital, 2-174 Edobashi, Tsu, Mie 514-8507, Japan

Supplementary figure (figure S)..………………………………………………………2

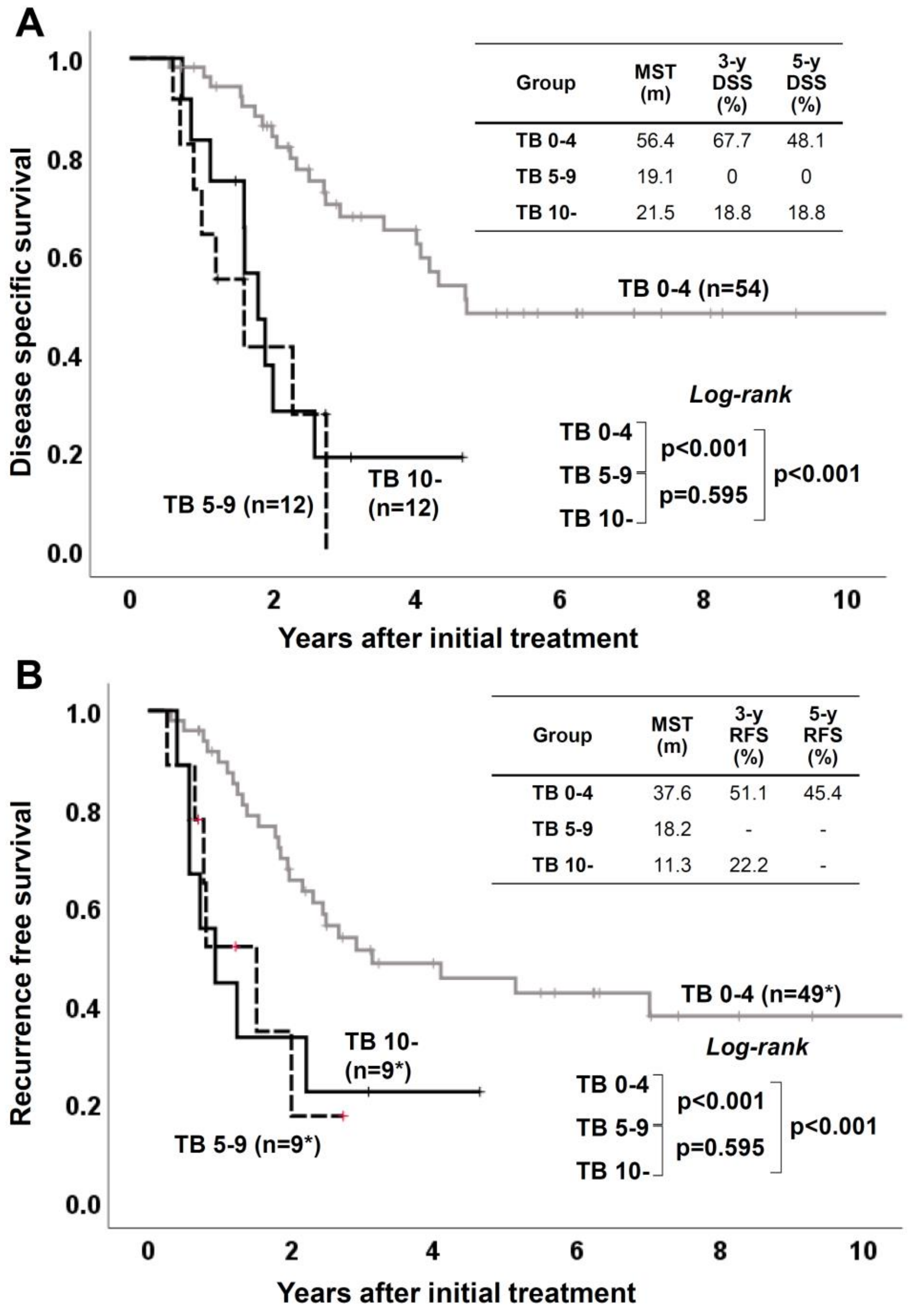

Figure S. Disease specific survival (DSS) and recurrence free survival (RFS) according to TB counts.

Both of DSS (A) and RFS (B) did not show differences between patients with TB 5-9 and those with TB 10 or more.
